# Effect of Three Months of Honey Supplementation on Heart Rate Variability and Baroreflex Sensitivity in Type 2 Diabetic Neuropathy Patients

**DOI:** 10.7759/cureus.88434

**Published:** 2025-07-21

**Authors:** Allampalli Sirisha, Girwar Singh Gaur, Pravati Pal, Suryanarayana B Shamanna, Zachariah Bobby, Gopal Krushna Pal

**Affiliations:** 1 Physiology, Sri Venkateswara Medical College, Tirupati, IND; 2 Physiology, Jawaharlal Institute of Postgraduate Medical Education and Research, Puducherry, IND; 3 General Medicine, Jawaharlal Institute of Postgraduate Medical Education and Research, Puducherry, IND; 4 Biochemistry, Jawaharlal Institute of Postgraduate Medical Education and Research, Puducherry, IND

**Keywords:** autonomic neuropathy, baroreflex sensitivity, heart rate variability, honey, type 2 diabetes

## Abstract

Background

Honey, by means of its hypoglycemic, hypolipidemic, and antioxidant properties, might help in preventing the progression of cardiovascular autonomic neuropathy in diabetes. In this study, we aimed to investigate the effect of honey supplementation for three months on cardiovascular autonomic function tests in diabetic neuropathy (DN) patients.

Methodology

This follow-up study was conducted among 48 type 2 diabetic patients who were on antidiabetic drugs for a minimum of one year. Heart rate variability (HRV), baroreflex sensitivity (BRS), and classical autonomic function tests were performed for all recruited participants pre- and post-honey supplementation. Honey was administered at a dose of 0.5 g/kg body weight/day for three months.

Results

After three months of honey supplementation, the systolic and diastolic blood pressure reduced significantly. The HRV parameters showed a significant increase in the square root of the mean squared differences of successive normal to normal intervals, standard deviation of normal to normal intervals, and total power, along with a reduction in the low-frequency component expressed as normalized unit and ratio of low-frequency power to high-frequency power of HRV. Further, the difference in diastolic blood pressure between sitting and the isometric hand grip procedure decreased, and the BRS increased significantly. There was a significant reduction in fasting blood glucose, lipid profile, and markers of oxidative stress. A significant rise in nitric oxide was noted after three months of honey supplementation.

Conclusions

Three months of honey supplementation in diabetic patients along with antidiabetic treatment helped in a substantial reduction in blood glucose, dyslipidemia, and oxidative stress parameters, which are markers of cardiovascular risk. There was attenuation of autonomic imbalance with augmentation of the vagal drive and a reduction in the sympathetic activity in these patients.

## Introduction

Diabetes mellitus is a prevalent non-communicable disease worldwide. According to the recent report from the International Diabetes Federation, diabetes had a global prevalence of about 537 million in 2021 [1]. Type 2 diabetes mellitus (T2DM), the more common type of diabetes, accounts for more than 90% of diabetes cases. Chronic hyperglycemia is the characteristic feature of T2DM, together with disturbances in carbohydrate, protein, and fat metabolism, which is the consequence of defects in insulin action, insulin secretion, or both [2]. Patients with diabetes have an increased incidence of atherosclerotic cardiovascular, retinal, cerebrovascular, and peripheral arterial disease [3].

More than 50% of diabetic patients develop diabetic neuropathy (DN) with long-standing T2DM [4]. Diabetic autonomic neuropathy (DAN) is the second most common type of neuropathy in the diabetic population, next to peripheral neuropathy [5]. Clinically, the most important form of DAN is cardiovascular autonomic neuropathy (CAN), which is associated with an increased rate of mortality and morbidity, in addition to several unfavorable outcomes [6]. CAN is caused by damage to the autonomic nerve fibers that innervate the heart. CAN is usually subclinical; an early sign of this condition is alteration in heart rate variability (HRV), which can be detected by using cardiovascular autonomic function tests (CAFTs) [7]. Due to the increased mortality rate, the treatment of CAN is challenging for clinicians. The recent report from the World Health Organization on non-communicable disease states that cardiovascular diseases (CVDs) are the primary reason for global deaths, with 17.9 million deaths per year [8]. In diabetics, there is increased CVD risk mainly because of persistent hyperglycemia, as well as other factors such as high blood pressure and abnormal cholesterol levels [9,10]. Despite adequate treatment with allopathic antidiabetic medicines, diabetes and its associated complications persist, emphasizing the need for a multifaceted approach that includes lifestyle modifications, regular monitoring, and possibly complementary therapies to achieve optimal management and reduce the risk of complications [11].

Honey, a naturally sweet substance with a history of traditional use, has been valued for its various beneficial properties for decades. It is reported to have several medicinal properties, including antioxidant, antibacterial, antihyperglycemic, hepatoprotective, and antihypertensive effects [12-14]. Natural honey contains flavonoids, amino acids, phenolic acids, and ascorbic acids, which are well known for their antioxidant activity [15]. Honey supplementation is reported to reduce high blood glucose values and hyperlipidemia in type 1 and 2 diabetes mellitus patients [16,17]. Studies on the antihypertensive activity of honey are relevant due to its antioxidant properties. Though studies have reported the cardioprotective effects of honey [18,19], most of these studies were conducted on animal models. Very few studies have been conducted among humans to identify the cardioprotective effects of honey. Further, there are limited studies on the long-term effects of honey in diabetic patients. To our knowledge, there are no studies demonstrating the long-term effects of honey on cardiovascular functions or autonomic imbalance in diabetic patients. Given the limited evidence in human populations, this study aimed to assess whether three months of daily honey supplementation could improve cardiovascular autonomic regulations evaluated by HRV and baroreflex sensitivity (BRS) as the primary outcomes, as well as reduce oxidative stress, dyslipidemia, and hyperglycemia as secondary outcomes in patients with type 2 DN.

## Materials and methods

The protocol was approved by the Scientific Advisory Committee of Jawaharlal Institute of Postgraduate Medical Education and Research (JIPMER) (approval number: JIP/IEC/2016/1142), and consent procedures were approved and followed as per the Institute Ethics Committee of JIPMER. All study participants were more than 18 years of age and provided written informed consent. This clinical trial was registered at the Clinical Trials Registry-India prospectively (registration number: CTRI/2017/09/009617).

Study design and participants

**Figure 1:**
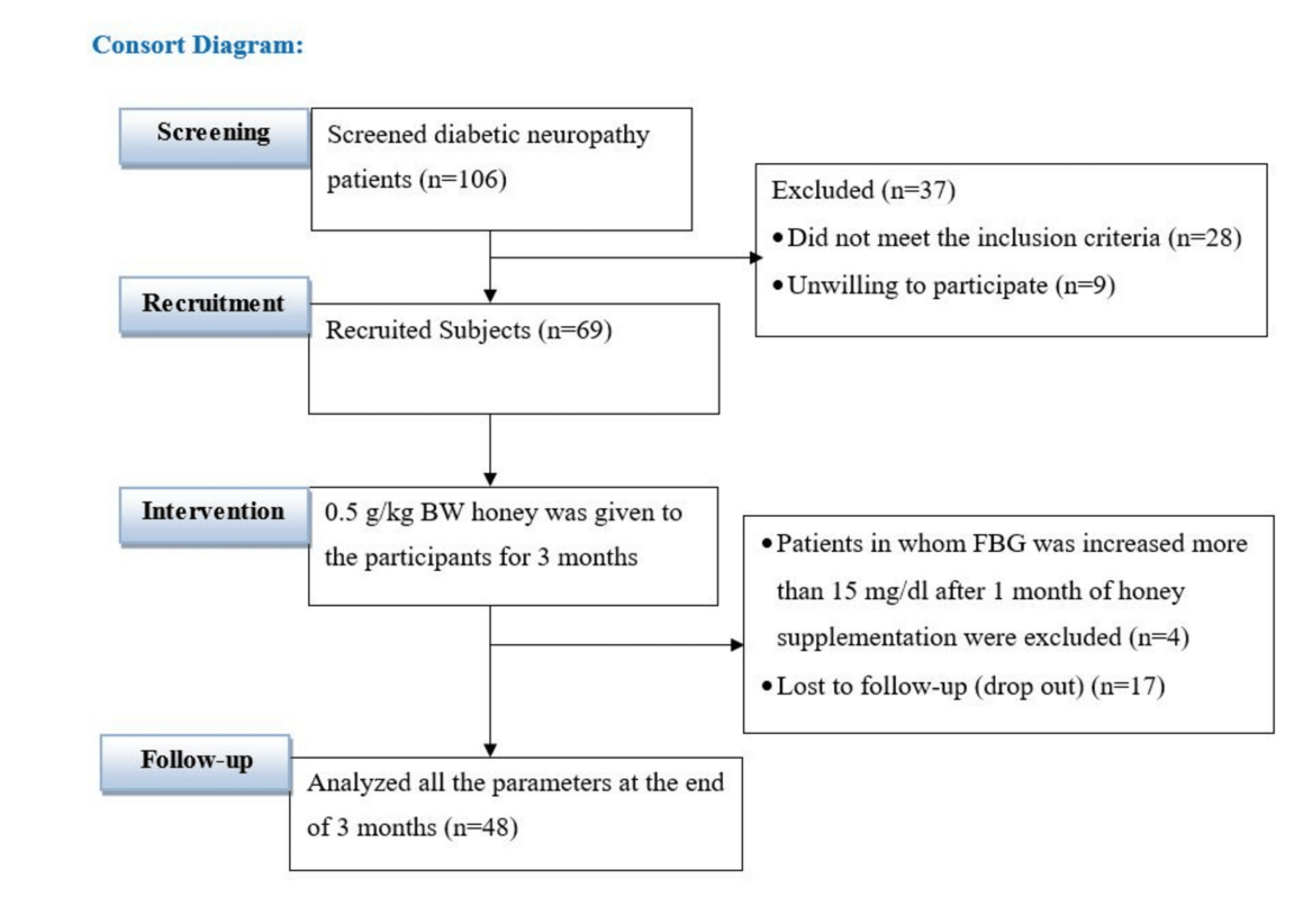
CONSORT diagram showing the recruitment and follow-up of participants.

This study was conducted at JIPMER, Puducherry, India. In this single-arm, open-label, pilot trial, we recruited individuals who were above 18 years of age, had T2DM for more than five years, were diagnosed clinically for neuropathy, and were on treatment for a minimum of one year. Participants were excluded from the trial if they presented with any other neurological problems, any chronic or acute ailments, any cardiac or renal diseases, or were on hormonal therapy. Given the challenge in recruiting pure diabetic individuals with a disease duration of over five years, the conditions of hypertension and hyperlipidemia were not excluded. However, they remained stable and were consistently managed throughout the study period. Importantly, as our outcome measurements were assessed within the same group before and after honey supplementation (pre-post design), the influence of these comorbidities as confounders was minimized. Participants were recruited into the study from the Diabetic Clinic, Department of Medicine, JIPMER. A total of 447 T2DM patients were screened for DN based on clinical diagnosis and inclusion and exclusion criteria. Following this, 106 DN patients were approached and asked to participate in this study. Of the 106 patients, 69 agreed to give written and informed consent and participated in the study. After three months of honey consumption along with regular treatment, 48 participants returned for final evaluation (Figure 1).

Honey intervention

Participants consumed honey daily along with their normal breakfast. Honey was consumed at a dose of 0.5 g/kg body weight per day. It was mixed with 250 mL of water for easy consumption. The dosage of honey was decided based on a previous study [20]. Honey samples were acquired from different parts of the country and tested for their fructose levels and oxidative properties. The honey samples were also tested for adulteration using the water test and ant test [21]. The samples from Green Planet Trust, Theni, Tamil Nadu, India, were selected for procurement, which was approved by the Food Safety and Standards Association of India. Three batches of honey were procured for the study from Green Planet Trust. According to the instructions provided earlier to the vendor, we received liquid chromatography-mass spectrometry analysis reports of honey with every batch. Though there are minute variations in the composition of honey, there were no significant differences in the fructose content and antioxidant properties of the honey from the three batches. The study outcomes were measured at baseline and three months after the honey intervention.

Measurements

Each participant reported to the Department of Physiology at around 8:00 AM on the dates prescribed during their outpatient visits. They were asked to fast overnight to collect a 5 mL fasting blood sample used for the estimation of biochemical parameters. Height and weight were measured to calculate body mass index (BMI). After 10 minutes of supine rest in the polygraph laboratory, baseline heart rate (HR) and blood pressure (BP) were recorded, followed by HRV, CAFT, and blood pressure variability (BPV). HRV recordings were performed using the BIOPAC MP 100 data acquisition system (BIOPAC Inc., USA). HRV was analyzed from the digitized analog lead-II ECG signals using Kubios software version 2.2, with a sampling frequency of 1,000 Hz and a standard artifact correction filter.

Cardiovascular autonomic function tests

Participants were instructed to avoid caffeine and physical activity before testing. The resting HRV was performed with loose, comfortable clothing in a quiet ambience and controlled lab temperature (24-26°C). CAFTs were performed in the following sequence: after a period of comfortable rest in the supine position for 10 minutes, HR and BP response to deep breathing, change in posture from supine to standing, and isometric handgrip were recorded. Each procedure was performed as per the standard procedure [22], with a period of comfortable rest after each recording. The BIOPAC MP 100 data acquisition system was used for recording ECG and respiratory tracings of CAFTs (BIOPAC Inc., USA).

Recording of beat-to-beat blood pressure variability indices

The continuous beat-to-beat BPV was measured using a non-invasive continuous hemodynamic monitor, Finapres (Finometer version 1.22a, Finapres Medical Systems BV, Amsterdam, the Netherlands). Continuous BP recording of the participants was obtained for over 10 minutes, following which the reconstructed brachial pressure was acquired via a PC-based data acquisition system. The major parameters recorded from the reconstructed brachial pressure tachogram were stroke volume (SV), left ventricular ejection time (LVET), cardiac output, total peripheral resistance (TPR), and BRS.

Biochemical parameters

The fasting blood glucose (FBG) and plasma lipids, including total cholesterol (TC), triglycerides (TGs), high-density lipoprotein-cholesterol (HDL-C), and low-density lipoprotein-cholesterol (LDL-C), were estimated using a fully automated clinical chemistry analyzer. The following formula was used to calculate the atherogenic index (AI): AI = (TC-HDL)/HDL. Other serum parameters such as nitric oxide (NO), total antioxidant status (TAS), and thiobarbituric acid reactive substance (malondialdehyde/MDA) were also estimated.

Statistical analysis

Statistical analysis was performed to compare the parameters before and after three months of honey supplementation. SPSS version 19 (IBM Corp., Armonk, NY, USA) was used for statistical analysis. The data were expressed as mean ± SD, and all the data were run through the Shapiro-Wilk normality test before analysis. Student’s paired t-test was used to examine intergroup differences in mean. Whenever the probability was less than 0.05, the difference was considered statistically significant.

## Results

A total of 48 study participants consumed honey for three months and presented for follow-up recordings. Overall, 35 males and 13 females were included, with a mean age of 58.91 ± 7.97 years. The average BMI of the study participants was 27.16 ± 3.79 kg/m^2^, with a mean HbA1c of 7.86% ± 1.39%. Three months of honey supplementation did not show any significant difference in body weight and BMI. Although a slight decrease was observed in HbA1c (7.59% ± 1.46%) after three months of honey supplementation, the reduction was not statistically significant.

Table 1 shows that basal cardiovascular parameters, namely, systolic blood pressure (SBP) (p = 0.0045), diastolic blood pressure (DBP) (p = 0.0399), and HR (p = 0.0030), reduced significantly after three months of honey supplementation. The calculated cardiovascular parameters, namely, mean arterial pressure (MAP) (p = 0.0043) and rate-pressure product (RPP) (p = 0.0001), also reduced significantly (Table 1).

**Table 1:** Comparison of anthropometric and basal cardiovascular parameters before and after three months of honey supplementation. Values are expressed as mean ± SD. Analysis was done by Student’s paired t-test. *: P-values less than 0.05 were considered statistically significant. TBW: total body weight; BMI: body mass index; HR: heart rate; SBP: systolic blood pressure; DBP: diastolic blood pressure; MAP: mean arterial pressure; RPP: rate-pressure product

Parameters	Before honey supplementation (n = 48)	After three months of honey supplementation (n = 48)	P-value
Age (years)	58.91 ± 7.976	-	-
TBW (g)	71.25 ± 11.75	70.75 ± 11.88	0.1647
BMI (kg/m^2^)	27.16 ± 3.79	27.01 ± 3.79	0.1773
SBP (mmHg)	133.05 ± 20.94	124.92 ± 14.47	0.0045*
DBP(mmHg)	74.42 ± 10.22	70.99 ± 8.34	0.0399*
HR (beats/minute)	82.01 ± 11.51	76.05 ± 9.91	0.0030*
MAP (mmHg)	93.96 ± 11.70	88.97 ± 8.62	0.0043*
RPP	110.02 ± 26.45	95.36 ± 18.60	0.0001*

Table 2 shows a comparison of HRV parameters, indicating a significant increase in the time domain indices of HRV such as mean R-to-R interval (mean RR) (p = 0.0070), root mean square of successive differences between normal heartbeats (RMSSD) (p = 0.0090), and standard deviation of normal-to-normal interval (SDNN) (p = 0.0110) after three months of honey supplementation. The frequency domain indices showed a significant increase in total power (TP) (p = 0.0002) and a significant reduction in the low frequency component expressed as normalized unit (LF nu) (p = 0.0382) and the ratio of low-frequency power to high-frequency power of heart rate variability (LF:HF) (p < 0.0001). The high frequency component expressed as normalized unit (HF nu) did not show a significant difference after honey supplementation (Table 2).

**Table 2:** Comparison of time domain and frequency domain indices of HRV parameters in patients before and after three months of honey supplementation. Values are expressed as mean ± SD. Analysis was done by Student’s paired t-test. *: P-values less than 0.05 were considered statistically significant. HRV: heart rate variability; Mean RR: mean RR interval; SDNN: standard deviation of normal-to-normal interval; RMSSD: square root of the mean squared differences of successive normal-to-normal intervals; TP: total power; HF: high frequency; LF: low frequency; VLF: very low frequency; LFnu: low frequency component expressed as the normalized unit; HFnu: high frequency component expressed as the normalized unit; LF-HF ratio: ratio of low-frequency power to high-frequency power of heart rate variability; DN: diabetic neuropathy

Parameters	Before honey supplementation (n = 48)	After three months of honey supplementation (n = 48)	P-value
Time domain indices			
Mean RR	746.59 ± 110.35	803.54 ± 116.37	0.0070*
RMSSD	26.87 ± 12.89	32.85 ± 14.07	0.0090*
SDNN	25.38 ± 9.98	31.13 ± 10.03	0.0110*
Frequency domain indices			
TP	370.16 ± 218.20	556.78 ± 258.92	0.0002*
LF nu	54.32 ± 22.51	46.40 ± 15.88	0.0382*
HF nu	47.06 ± 20.58	51.51 ± 17.27	0.2613
LF:HF	2.49 ± 1.54	1.36 ± 0.77	<0.0001*

The CAFT parameters are presented in Table 3. The ratio between the maximum RR interval at the 30th beat and minimum RR interval at the 15th beat (30:15 ratio), the ratio of the longest RR interval during expiration to the shortest RR interval during inspiration averaged over six cycles of respiration (E:I ratio) were not significantly altered after three months, whereas difference in DBP between supine and isometric hand grip (∆DBP_^IHG^_) showed a significant reduction (p = 0.0095) in these patients (Table 3).

**Table 3:** Comparison of cardiovascular autonomic function test parameters before and after three months of honey supplementation. Values are expressed as mean ± SD. Analysis was done by Student’s paired t-test. *: P-values less than 0.05 were considered statistically significant. 30:15 ratio: ratio between the maximum RR interval at the 30th beat and the minimum RR interval at the 15th beat; E:I ratio: ratio of the longest RR interval during expiration to the shortest RR interval during inspiration averaged over six cycles of respiration; ∆ DBP_^IHG^_: difference in diastolic blood pressure between sitting and isometric hand grip

Parameters	Before honey supplementation (n = 48)	After honey supplementation (n = 48)	P-value
30:15	1.20 ± 0.22	1.19 ± 0.21	0.8203
E:I	1.25 ± 0.29	1.36 ± 0.31	0.0848
ΔDBP_IHG_	17.75 ± 5.62	14.85 ± 4.67	0.0095*

Post-honey supplementation LVET (p = 0.0311) and CO (p = 0.0115) reduced significantly, and BRS increased significantly (p < 0.0001). However, SV and TPR did not show any significant difference (Table 4).

**Table 4:** Comparison of blood pressure variability parameters in patients before and after three months of honey supplementation. Values are expressed as mean ± SD. Analysis was done by Student’s paired t-test. *: P-values less than 0.05 were considered statistically significant. SV: stroke volume; LVET: left ventricular ejection time; PI: pulse interval; CO: cardiac output; BRS: baroreceptor reflex sensitivity; TPR: total peripheral resistance

Parameters	Before honey supplementation (n = 48)	After three months of honey supplementation (n = 48)	P-value
SV (mL/minute)	80.26 ± 18.25	73.20 ± 20.87	0.0870
LVET (ms)	319.30 ± 25.58	313.67 ± 21.85	0.0311*
CO (L/minute)	6.86 ± 1.77	5.85 ± 2.06	0.0115*
BRS (ms/mmHg)	5.4 ± 2.56	9.82 ± 4.47	<0.0001*
TPR (mmHg.minute/L)	0.71 ± 0.24	0.65 ± 0.32	0.2335

**Figure 2:**
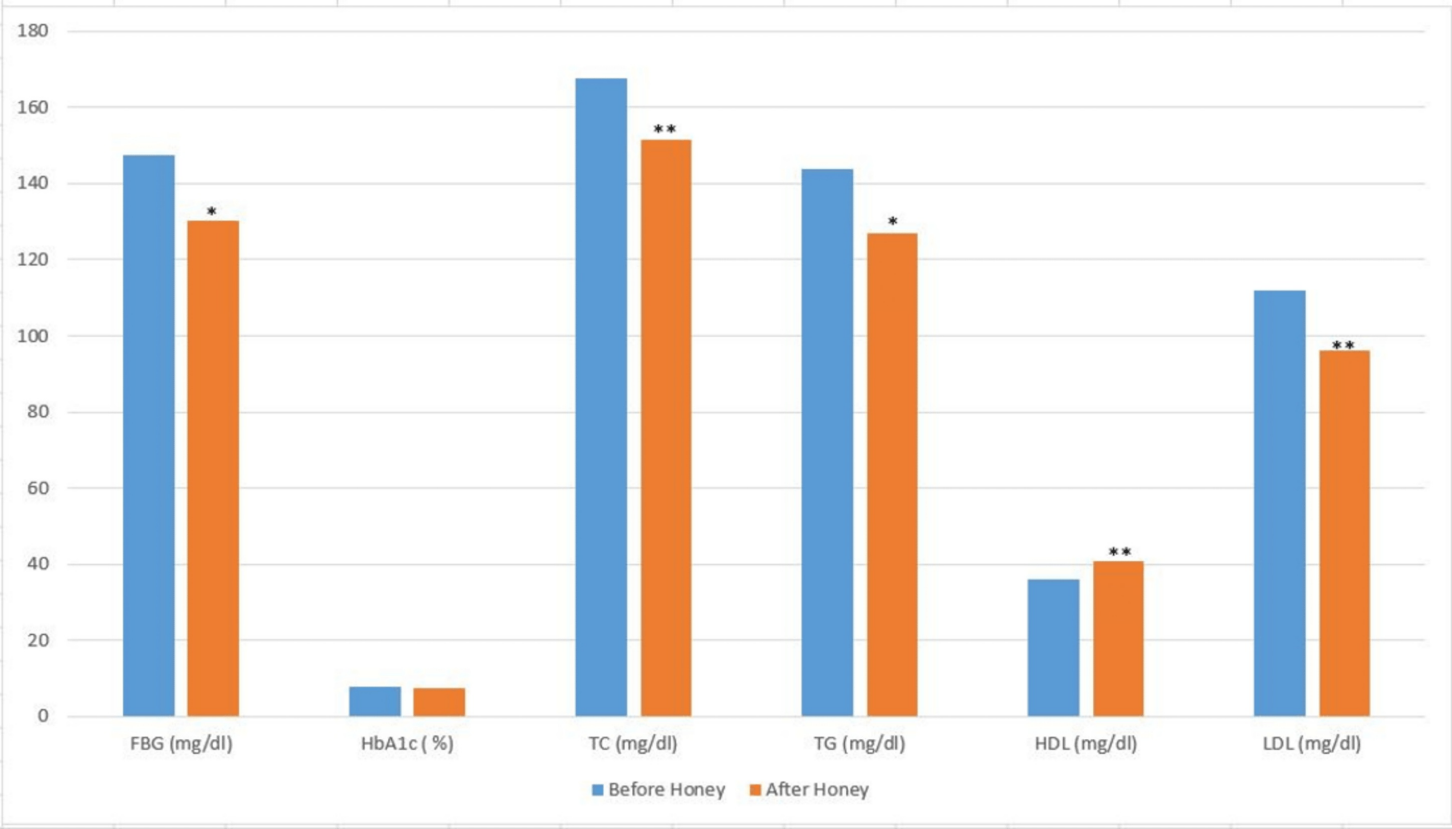
FBG and lipid parameters in patients before and after honey supplementation. Analysis was done by Student’s paired t-test. *: Comparison with before honey supplementation: *: p < 0.05; **: p < 0.01. FBG: fasting blood glucose; TC: total cholesterol; TG: triglycerides; HDL-C: high-density lipoprotein-cholesterol; LDL-C: low-density lipoprotein-cholesterol; HbA1C: glycated hemoglobin

Figure 2 shows biochemical parameters after three months of honey supplementation. A significant reduction was observed in FBG (p = 0.0192), but there was no significant difference in HbA1c. The lipid profile parameters, such as TG (p = 0.0371), TC (p = 0.0049), and LDL-C (p = 0.0060), were reduced significantly, and HDL-C (p = 0.0030) increased significantly after three months of honey supplementation (Figure 2).

In oxidative stress parameters, MDA was reduced (p = 0.0063) and TAS was increased (p < 0.0001) significantly after three months of honey supplementation. NO (p = 0.0138) increased significantly after three months of honey supplementation (Table 5).

**Table 5:** Comparison of biochemical markers before and after three months of honey supplementation. Values are expressed as mean ± SD. Analysis was done by Student’s paired t-test. *: P-values less than 0.05 were considered statistically significant. MDA: malondialdehyde; TAS: total antioxidant status

Parameters	Before honey supplementation (n = 48)	After honey supplementation (n = 48)	P-value
MDA (ng/mL)	24.48 ± 7.61	20.33 ± 6.62	0.0063*
TAS (μmol/L)	632.01 ± 177.25	768.69 ± 172.14	<0.0001*
Nitric oxide (µmol/L)	17.14 ± 5.00	20.04 ± 7.61	0.0138*

## Discussion

In the present study, the effect of three months of honey supplementation on cardiovascular autonomic functions was studied in diabetic patients. To eliminate the effect of antidiabetic treatment on the primary outcome of the study, newly diagnosed patients were not included in the study. Patients who were on treatment for diabetes for more than five years and on treatment for neuropathic complications for more than one year were included in the study. The average duration of neuropathic complications (with treatment) of the participants in the study was 2.79 ± 1.83 years.

Clinically, the most important autonomic neuropathy is CAN, owing to its associated increased mortality rate [23]. In diabetes, the release of advanced glycation end products due to persistent hyperglycemia might be a key factor in damaging cardiac neurons and consequent autonomic abnormalities, which lead to autonomic imbalance, i.e., between parasympathetic and sympathetic activity [24]. The parasympathetic and sympathetic neurons, which supply the heart and blood vessels, are most affected in diabetes, causing abnormalities in HR control and cardiovascular dynamics [25]. Honey is reported to reduce cardiovascular risk in obese and diabetic subjects [26,27]. However, these reports are based on the hypolipidemic and anti-inflammatory effect of honey, not on the assessment of the effect of honey on cardiovascular autonomic function. HRV analysis, CAFT, and BRS testing are well-established, non-invasive tests for identifying the cardiovascular risk and autonomic dysfunctions [7,28]. In the present study, the effect of honey on cardiovascular autonomic function was studied by using these standard tests.

In a study conducted by Olusola et al., honey consumption reduced BP and HR in normal individuals [21], which is in concurrence with our data showing a reduction in SBP, DBP, and HR (Table 1) after three months of honey supplementation in DN patients. Our data showed a significantly reduced LF-HF ratio (p < 0.0001) post-honey supplementation (Table 2). The LF-HF ratio is a profound marker of autonomic balance, indicating sympathetic over-activity and reduced vagal drive [29]. Reduced LF-HF ratio after honey supplementation indicates reduced sympathetic activity, which is further supported by a significant reduction in LF nu (p = 0.0382), as an increase in LF nu mainly represents heightened sympathetic discharge to the heart [30]. The present data also showed a significant increase in TP of HRV (p = 0.0002) after three months of honey supplementation (Table 2), demonstrating a substantial increase in the magnitude of HRV and vagal modulation of cardiac activities, as TP generally represents the effectiveness of the parasympathetic system on cardiac modulation [29,30]. In addition, a significant increase in all time-domain indices (TDIs) of HRV (mean-RR, SDNN, and RMSSD) (Table 2) further substantiates the augmented vagal modulation of cardiac functions, as TDIs are markers of parasympathetic modulation of heart functions [29,30]. Among TDIs, the RMSSD better represents vagal control of HR recorded on a short-term basis and is a significant marker of parasympathetic drive to the heart [30]. Increased RMSSD post-honey supplementation reflects the enormity of improved vagal tone in these subjects, which is further substantiated by a significant increase in BRS after honey supplementation (Table 4), as BRS is an indicator of parasympathetic activity [28,31]. Following three months of honey supplementation, RPP reduced significantly (Table 1), indicating the reduction in myocardial oxygen demand and utilization, as RPP is a major determinant of myocardial workload and cardiac oxygen consumption [32]. The marker of sympathetic reactivity test, i.e., ∆DBP_IHG_ showed a significant decrease (Table 3) after three months of honey supplementation in these patients, indicating reduced sympathetic reactivity [33].

Although the exact mechanism of attenuation of autonomic imbalance by honey is not known, it might be due to the anti-hyperglycemic and anti-lipidemic effects of honey along with its antioxidant properties. In our study, there was a significant reduction in FBG after three months (Figure 2), confirming the anti-hyperglycemic effect of honey in DN patients. There are very few reports on the effects of honey supplementation in patients with diabetes, especially for longer durations [34,35]. Our study findings on FBG are in concordance with these reports. Along with persistent hyperglycemia, lipotoxicity is one of the reasons for cardiovascular dysfunctions in diabetes [36]. In the present study, plasma lipids were reduced significantly, except HDL-C, the good cholesterol, which increased significantly after three months of honey supplementation, confirming the hypolipidemic effect of honey in diabetic patients. Similarly, other studies have reported the hypolipidemic effect of honey in patients with diabetes and normal individuals [17,37]. Increased lipid peroxidation is caused by lipid accumulation. The end products of this lipid peroxidation enhance the formation of free radicals, which lead to increased oxidative stress in diabetic patients. By improving lipid metabolism and reducing oxidative stress, honey is known to reduce cardiovascular risk [34,38]. After three months of honey supplementation, there was a significant reduction in MDA and a significant increase in TAS (Table 5), indicating the protective effects of honey on oxidative damage in DN patients. Though studies in diabetic rats reported honey as a potent antioxidant [12,14,39], to our knowledge, there are limited studies in humans on the effect of honey on antioxidant status.

**Figure 3:**
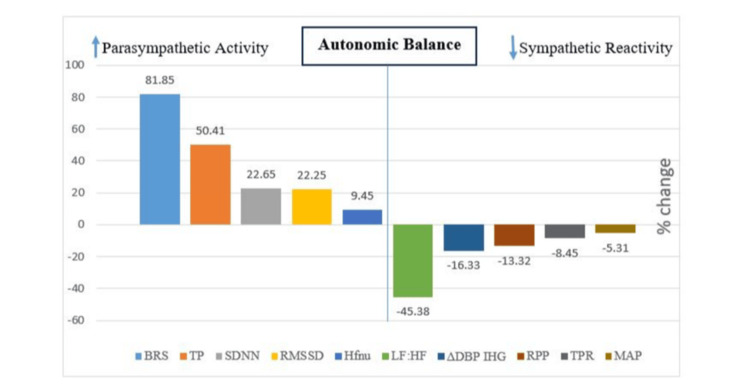
Percentage changes in autonomic function parameters in patients after three months of honey supplementation. BRS: baroreceptor reflex sensitivity; SDNN: standard deviation of normal-to-normal interval; RMSSD: square root of the mean squared differences of successive normal-to-normal intervals; HFnu: high frequency component expressed as normalized unit; TP: total power; LF-HF ratio: ratio of low-frequency power to high-frequency power of heart rate variability; ∆DBPIHG: Difference in diastolic blood pressure between sitting and isometric hand grip; TPR: total peripheral resistance; RPP: rate-pressure product; MAP: mean arterial pressure

The present report of honey supplementation attenuating autonomic imbalance is further substantiated by our data showing a significant increase in NO level following three months of honey supplementation (Table 5) in diabetic patients. This increased NO indicates the improvement in autonomic imbalance, as NO is a potent vasodilator, which protects the autonomic nervous system by increasing the vagal tone and decreasing the sympathetic tone. However, it is to be noted that NO can be influenced by diet, inflammation, or physical activity. Studies have reported that honey contains NO metabolites, and it increases NO production in various biological fluids [40], which was also observed in our study. The current report of the effect of honey on attenuation of autonomic imbalance by enhancement of parasympathetic tone and reduction in sympathetic activity (Figure 3) is the first report of its kind, as no similar reports have shown the effect of honey on autonomic functions in humans.

Study limitations

Per the safety aspect under ethical recommendation, four patients whose FBG increased more than 15 mg/dL were excluded from the study. The reason for their increase in FBG could not be assessed in this study.

## Conclusions

From the findings of this study, we infer that three months of honey supplementation to T2DM patients, along with antidiabetic treatment, helps in a substantial reduction in blood glucose, dyslipidemia, and oxidative stress parameters, which are markers of cardiovascular risk. This cardioprotective effect of honey helped in attenuation of autonomic imbalance, which is shown by augmentation of the vagal drive and reduced sympathetic overactivity in these patients. However, a follow-up study in a larger cohort with a standard control group would establish the amelioration of oxidative stress and lipotoxicity and the improvement in autonomic function in these patients.
